# Drought and heatwave synergy alters organ-level C:N stoichiometry and carbohydrate dynamics in subtropical tree seedlings

**DOI:** 10.3389/fpls.2026.1814780

**Published:** 2026-03-31

**Authors:** Zijun Deng, Shuhui Wang, Ran Zhang, Xinghao Tang, Yixuan Liu, Jiayi Lin, Youyou Zhang, Yalin Hu, Xiao-Li Yan, Lu-Ping Qu

**Affiliations:** 1 College of Forestry, Fujian Agriculture and Forestry University, Fuzhou, China; 2 Fujian Academy of Forestry Science, Fuzhou, China

**Keywords:** combined stress, legacy effects, metabolic debt, non-structural carbohydrates, source-sink coordination, subtropical tree species

## Abstract

**Introduction:**

Frequency and intensity of “hot droughts” are escalating globally, yet the physiological mechanisms governing tree resilience to the synergistic impacts of water deficiency and heatwaves remain poorly understood.

**Methods:**

Here, we investigated the dynamics of carbon (C), nitrogen (N), and non-structural carbohydrates (NSC) in leaves, stems, and roots of three subtropical tree seedlings (*Schima superba*, *Cyclobalanopsis glauca*, and *Phoebe bournei*) during combined stress and subsequent rehydration.

**Results:**

Our results demonstrate that combined stress triggers a “dual-deficit” mechanism—simultaneous cessation of carbon assimilation and accelerated respiratory consumption, as inferred from the significant depletion of organ-specific NSC pools—that exerts a more profound impact on NSC pools than on C-N stoichiometry. Structural equation modeling revealed a spatiotemporal division of labor where seedlings prioritize acute hydraulic safety by sacrificing foliar metabolic performance, incurring a delayed “metabolic debt” in roots that manifests as significant legacy effects during recovery.

**Discussion:**

Crucially, the three species exhibited divergent strategies to manage this metabolic capital: *S. superba* displayed high resilience through “physiological re-priming,” rapidly restoring foliar source capacity; *C. glauca* adopted a “conservative buffering” strategy, sequestering NSC in stems to bridge the gap between resistance and hydraulic recovery; conversely, *P. bournei* suffered from a maladaptive “root-leaf decoupling,” where structural rigidity in roots impeded the remobilization of reserves, leading to systemic NSC depletion indicative of potential carbon starvation. These findings underscore that resilience to compound climate extremes depends not merely on static resistance traits, but on the flexibility of inter-organ coordination to resolve the metabolic debts of recovery.

## 1 Introduction

As global climate change intensifies, the increasing frequency of abiotic stressors—particularly extreme heat and drought—threatens to disrupt plant carbon (C) and nitrogen (N) metabolism and internal resource balance, thereby compromising fundamental physiological functions (Dusenge et al., 2018; Grossiord et al., 2018; IPCC, 2023). Among these, drought remains a pervasive stressor, impairing a suite of physiological processes that govern tree growth and productivity (Mcdowell et al., 2016; Groover et al., 2025). Concurrently, extreme heat events, or heatwaves, are occurring with greater frequency and intensity worldwide (Kornhuber et al., 2024). In subtropical regions, persistent high-pressure systems often couple these heatwaves with drought, exacerbating the intensity of these co-occurring events (Zscheischler et al., 2020; Qu et al., 2024a). Such “dry-heat” stress induces synergistic negative impacts, including shoot dieback (Qu et al., 2020), reduced photosynthetic capacity (Zahra et al., 2023), and severe hydraulic failure (Gong and Hao, 2023). These pressures lead to increased regional vegetation mortality (Sato et al., 2024) and heighten the risk of plant community decline (Wolf and Paul-Limoges, 2023). Despite extensive research on individual thermotolerance and drought resistance (Geange et al., 2021; Guadarrama-Escobar et al., 2024; Ning et al., 2024), a systematic understanding of the physiological mechanisms underlying tree responses to combined drought and heatwave stress in subtropical ecosystems remains a significant challenge (Armstrong et al., 2023).

Carbon and nitrogen distribution represents a core physiological strategy for acclimating to such abiotic extremes. The carbon pool, partitioned into structural components and non-structural carbohydrates (NSC), modulates physical integrity and metabolic resilience (Piper and Paula, 2020; Rehschuh et al., 2021; Luo et al., 2024). Structural carbon investments—characterized by enhanced lignification and increased leaf mass per area (LMA)—fortify physical integrity, thereby minimizing desiccative water loss and protecting the photosynthetic apparatus from thermal degradation (Wang et al., 2025a). Concurrently, NSCs (primarily soluble sugars and starch) function as both essential osmoticums and mobile energy reservoirs (Dietze et al., 2014; Wang and Wang, 2025). Under drought, trees preferentially allocate NSCs to root systems to increase the root-to-shoot ratio, facilitating deep-water acquisition and metabolic survival (Guo et al., 2020; Hartmann et al., 2020). Conversely, heat stress triggers rapid NSC mobilization to fuel the energetic costs of repairing heat-damaged proteins and membranes (Gong and Hao, 2023). Similarly, nitrogen dynamics and organ-specific N allocation are critical for maintaining homeostasis (Zhang et al., 2020; Ru et al., 2023). Drought-induced N reallocation typically prioritizes foliar N for osmotic adjustment and protective protein synthesis (Iqbal et al., 2020), often at the expense of stem N reserves (Meng et al., 2016; Zahoor et al., 2017). Root N dynamics—highly sensitive to N forms—are also pivotal, with ammonium often conferring superior drought resilience compared to nitrate (Sun et al., 2025). Regarding thermotolerance, optimal N nutrition enhances heat resistance by activating signaling modules (e.g., the NLP3-HSF pathway) that upregulate heat shock proteins (Wang et al., 2022; Zhu et al., 2025), while optimizing intra-leaf N partitioning to balance carbon fixation with photoprotection (Hou et al., 2019; Cun et al., 2021). The convergence of C and N metabolic shifts is ultimately reflected in the carbon-to-nitrogen ratio (C:N ratio, hereafter abbreviated as CNR), an integrative indicator of organ-specific resource stoichiometry (Qin et al., 2025). Plants enhance survivability by actively modulating CNR; for instance, increasing root CNR through prioritized C allocation supports root expansion during water deficits (Sevanto et al., 2014) while maintaining foliar CNR stability is essential for sustaining the enzymatic machinery required for N-based defense under high temperatures (Hartmann et al., 2018; Adams et al., 2020). This proactive regulation of C, NSC, and N concentrations underscores a sophisticated adaptive plasticity, shifting metabolic pathways from growth-oriented to defense-oriented investment (McDowell et al., 2022). However, most current research evaluates these regulatory strategies under isolated stress factors. Given that the physiological responses to heat and drought are often compensatory or even contradictory (Sato et al., 2024), the mechanisms governing C-N coupling and the resulting adaptive strategies under combined stress conditions remain poorly understood.

Furthermore, plant resilience to episodic drought or heatwaves comprises two distinct yet interdependent phases: resistance during the stress event and subsequent recovery (Ingrisch and Bahn, 2018; Qu et al., 2024a; Wang et al., 2025b). Resilient plants must not only ensure survival under extreme conditions (resistance) but also sequester sufficient “metabolic capital” to fuel regeneration once conditions ameliorate (Lalor et al., 2023; O’Connell and Wiley, 2024). According to resource trade-off theory, a physiological “debt” may be incurred if plants exhaust substantial C and N reserves to maintain immediate survival, potentially compromising the rate and magnitude of post-stress recovery (Li et al., 2020; Rehschuh et al., 2022). Conversely, strategic resource conservation during the stress period may facilitate more rapid compensatory growth afterward (Ruehr et al., 2019). Consequently, characterizing C and N metabolism solely during the stress period is insufficient for a comprehensive assessment of resilience. It is imperative to evaluate the dynamic reallocation of resources during the recovery phase (Rehschuh et al., 2022). Such post-stress adjustments are critical for repairing cellular damage and mitigating the “legacy effects” of stress, thereby fortifying the plant’s adaptive capacity against recurrent environmental challenges.

*Schima superba* (Theaceae), *Phoebe bournei* (Lauraceae), and *Cyclobalanopsis glauca* (Fagaceae) are co-occurring dominant canopy species in China’s subtropical evergreen broadleaf forests, representing significant ecological and economic value (Bruelheide et al., 2014; Xiong et al., 2023). Despite high annual precipitation in these regions, intense summer evapotranspiration often imposes severe evaporative demand, making water availability a critical limiting factor (Sanginés de Cárcer et al., 2018). The monsoon climate frequently precipitates concurrent drought and heatwaves—forming a synergistic “dry-heat” stress—that impairs biomass accumulation and destabilizes the carbon-water balance of subtropical vegetation (Wang et al., 2023; Xu et al., 2024). Consequently, quantifying organ-specific shifts in C and N stoichiometry is essential for deciphering the metabolic partitioning and adaptive strategies these species employ to navigate increasingly stressful habitats.

In this study, we investigated organ-specific C and N dynamics in *S. superba*, *P. bournei*, and *C. glauca* during acute stress (one day after rewatering, D1) and after a 30-day recovery period (D30) following drought, heatwave, and combined stress treatments. We hypothesized that: (1) combined drought and heat stress will exert a synergistic negative impact on C and N homeostasis, leading to a more profound depletion of NSC reserves and sharper CNR fluctuations compared to single stressors; (2) to enhance resistance, species will exhibit organ-specific C/N modulation—specifically, prioritizing C allocation to roots during drought for water foraging, while mobilizing NSC to leaves under heat stress for thermal repair; (3) recovery potential will be determined by the “metabolic capital” preserved during stress, where species maintaining stable C/N ratios and higher NSC legacies will demonstrate superior compensatory growth; and (4) these three species will display divergent adaptive strategies in C-N coupling, reflecting distinct trade-offs between structural investment (persistence) and metabolic flexibility (recovery). To test these hypotheses, we conducted a controlled experiment to quantify dynamic changes in C and N concentrations, NSC pools, and CNR across various organs, followed by a post-stress recovery assessment.

## 2 Materials and methods

### 2.1 Experimental site overview

The experiment was conducted at the Nursery of the Fujian Academy of Forestry Sciences (26°9′2″N, 119°17′2″E; 153.9 m a.s.l.). The site has a typical subtropical monsoon climate, with an average annual precipitation of 1342 mm, a mean annual temperature of 19.6 °C, and annual sunshine duration of 1700–1900 h, providing suitable conditions for seedling experiments.

### 2.2 Experimental materials

We used two-year-old seedlings of *Schima superba* (*S. superba*), *Phoebe bournei* (*P. bournei*), and *Cyclobalanopsis glauca* (*C. glauca*, obtained from the Fujian Academy of Forestry Sciences) to represent dominant subtropical broadleaf species sensitive to early growth conditions. In April of the experimental year (2023), we selected seedlings with uniform size (height: 30 ± 5 cm; basal diameter: 3 ± 0.5 mm), intact root systems, and no visible disease, and transplanted them into pots (44 cm × 29 cm) to avoid pot-bound effects. The growth medium consisted of a 1:1 mixture of local red soil (collected from a hillside at 500 m elevation near Fuzhou; total organic C: 47.3 g·kg^−^¹, total N: 3.2 g·kg^−^¹, pH 4.9) and a commercial substrate (imported peat, coconut coir, perlite, and controlled-release fertilizer; organic matter ≥ 30%, N+P_2_O_5_+K_2_O ≥ 3%, C/N < 25). This mixture aimed to simulate natural soil properties while ensuring adequate drainage and aeration. Each pot was filled with 10 kg of the medium. Seedlings were acclimatized under natural conditions with routine irrigation until the start of the stress treatments.

### 2.3 Experimental design

The experiment comprised four treatments: Control (C), Water Deficiency (D), Heat Wave (H), and Combined Water Deficiency + Heat Wave (DH), each applied within a separate semi-open transparent greenhouse. Each greenhouse contained 24 pots, with eight replicates per tree species arranged randomly. In the Control treatment, plants were grown under ambient conditions with natural rainfall. A transparent shelter (without heating) was installed during the heatwave period to control for shading effects, and soil moisture was maintained at 35–40% volumetric water content (SWC) via supplemental irrigation.

The Water Deficiency treatment was initiated on August 7 by covering the soil surface in the designated greenhouses (D and DH) with plastic film to exclude rainfall (**Supplementary Figure 1**). Soil moisture, measured daily using time-domain reflectometry (TDR), was allowed to decline gradually from field capacity (~35–40% SWC) to a target severe drought level of approximately 15% SWC by the end of August, simulating a progressive soil drying cycle.

The Heatwave treatment was simulated from September 2 to 6 (5 days) in greenhouses assigned to the H and DH groups. Each greenhouse was a semi-open, transparent structure (3 m × 3 m × 2 m, hollow steel frame covered with PVC film) featuring openable windows on all sides to maintain air circulation. A 3500 W industrial fan heater (BGE, Germany) was suspended at a height of 1.5 m and fixed at a 70° angle to avoid direct hot air exposure to seedlings; it was connected to a digital temperature controller. The controller was set to maintain the air temperature at 40 ± 2 °C during the daytime (09:00–17:00), based on recorded extreme heatwave temperatures in Fujian (**Supplementary Figure 1**). Transparent shelters were installed uniformly over all plots before the simulation to standardize structural effects. To ensure adequate soil moisture, seedlings were watered thoroughly one day prior to the experiment and manually irrigated as needed during the simulation to maintain target moisture levels. This heating regime successfully maintained air temperatures between 35 °C and 40 °C, resulting in an average air temperature increase of 7.1 °C and an average soil temperature increase of 3–5 °C relative to the Control, while soil moisture decreased by approximately 6%. Rainfall was blocked with plastic film in the heated greenhouses during this period. No significant differences in environmental indicators persisted among treatments after the simulation concluded.

All stress treatments were terminated on September 6, after which all plants were rewatered to field capacity. Destructive harvesting was conducted at 1 day (September 7) and 30 days (October 6) after rewatering. At each harvest time and for each treatment, four independent seedling replicates per species (n=4) were randomly selected. Roots, stems, and leaves were collected separately for subsequent analysis.

### 2.4 Sample collection and analysis

Seedlings were destructively sampled and separated into roots, stems, and leaves. Samples were blanched at 105 °C for 30 minutes to arrest metabolic activity and subsequently dried at 65 °C to a constant weight. Dried tissues were ground into a fine powder using a ball mill (Shanghai Jingxin, China). Total carbon (TC) and total nitrogen (TN) contents were determined using an elemental analyzer (Elementar Vario EL, Germany). Non-structural carbohydrates (NSC), including soluble sugars and starch, were quantified using the anthrone-sulfuric acid colorimetric method and perchloric acid hydrolysis, respectively.

### 2.5 Data analysis

All data are presented as the mean ± standard error (SE) of four biological replicates (independent seedlings). To assess the main and interactive effects of species, treatment, organ, and sampling time on each response variable (C, N, CNR, and NSC), a four-way factorial analysis of variance (ANOVA) was conducted using the general linear model (GLM). Where ANOVA indicated significant effects (α = 0.05), Duncan’s honestly significant difference (HSD) test was applied for *post-hoc* comparisons of treatment means within specific species-organ-time combinations. Duncan’s multiple range test was selected due to its higher sensitivity in pairwise comparisons—particularly when multiple treatment groups are contrasted with a single control—which aligns perfectly with the design of this study. To reduce collinearity among the correlated traits and to derive an integrated metric of plant metabolic status, a principal component analysis (PCA) was performed on four key traits—total carbon (TC), total nitrogen (TN), C:N ratio (CNR), and non-structural carbohydrate (NSC) content—measured separately in leaves, stems, and roots. The first principal component (PC1), which captured the largest proportion of variance, was extracted for each organ to serve as a composite metabolic index. Subsequently, a piecewise structural equation modeling (SEM) was constructed to analyze the direct effects of water deficiency, heatwave, and their interaction on these organ-specific composite indices. Within this framework, three separate linear models were specified, using the PC1 scores for leaves, stems, and roots as response variables and the three stress factors as predictors. These component models were subsequently integrated to evaluate the overall network of stress effects. Model fit was assessed using Fisher’s C statistic, where a non-significant p-value (*P* > 0.05) indicates good agreement between the model and the data. Standardized path coefficients, representing the strength and direction of effects, were derived from the component models. All analyses were performed in R (version 4.3.2) using the piecewiseSEM (v3.3.0), *nlme*, and *lme4* packages.

## 3 Results

### 3.1 Changes in C, N, NSC concentration and C:N ratio in different plant organs during rehydration following heatwave and water deficiency stress

**Figure 1:**
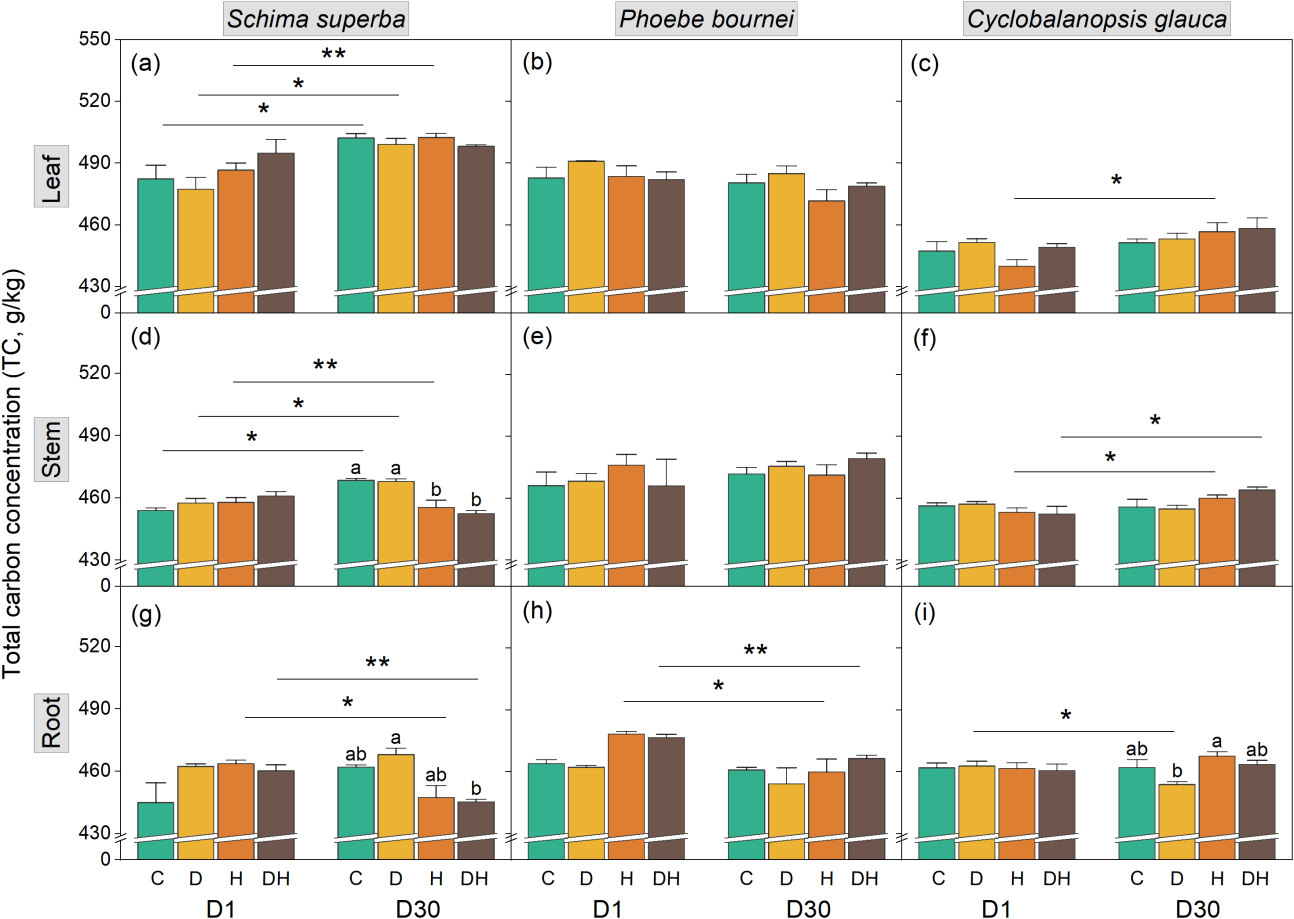
Dynamics of total carbon concentration (TC) in leaf **(a-c)**, stem **(d-f)**, and root **(g-i)** tissues of three subtropical tree species during rehydration. Seedlings of Schima superba, Phoebe bournei, and Cyclobalanopsis glauca were analyzed at 1 day (D1) and 30 days (D30) after rewatering following stress treatments. Treatments: C, control; D, water deficiency; H, heatwave; DH, combined stress. Different lowercase letters indicate significant differences between treatment groups within the same time point (P < 0.05). Asterisks denote significant differences for a specific treatment between D1 and D30 (*P < 0.05; **P < 0.01; ***P < 0.001).

**Figure 2:**
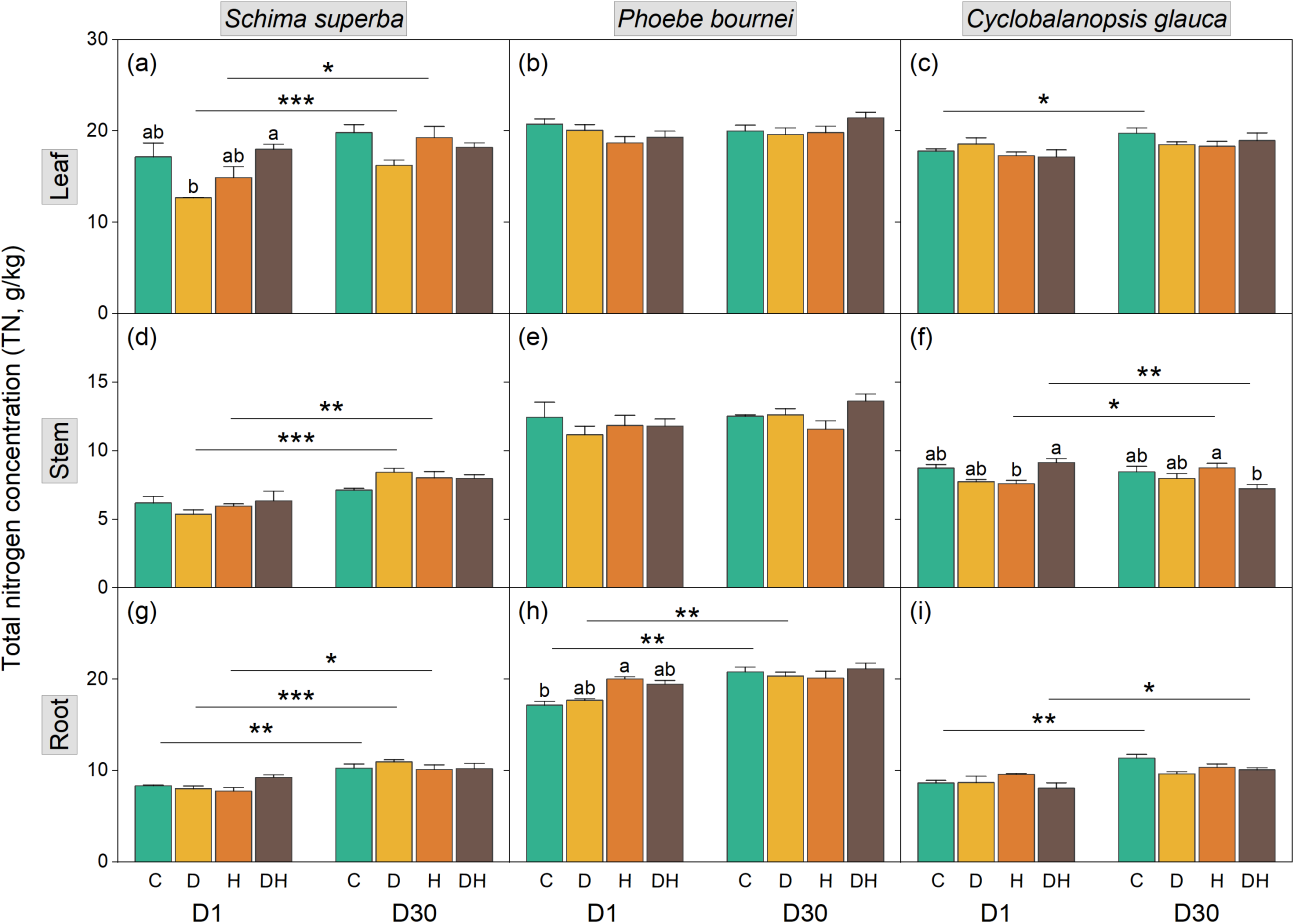
Dynamics of total nitrogen concentration (TN) in leaf **(a–c)**, stem **(d–f)**, and root tissues **(g–i)** of three subtropical tree species during rehydration. Seedlings of *Schima superba*, *Phoebe bournei*, and *Cyclobalanopsis glauca* were analyzed at 1 day (D1) and 30 days (D30) after rewatering following stress treatments. Treatments: C, control; D, water deficiency; H, heatwave; DH, combined stress. Different lowercase letters indicate significant differences between treatment groups within the same time point (*P* < 0.05). Asterisks denote significant differences for a specific treatment between D1 and D30 (*P < 0.05; **P < 0.01; ***P < 0.001).

**Figure 3:**
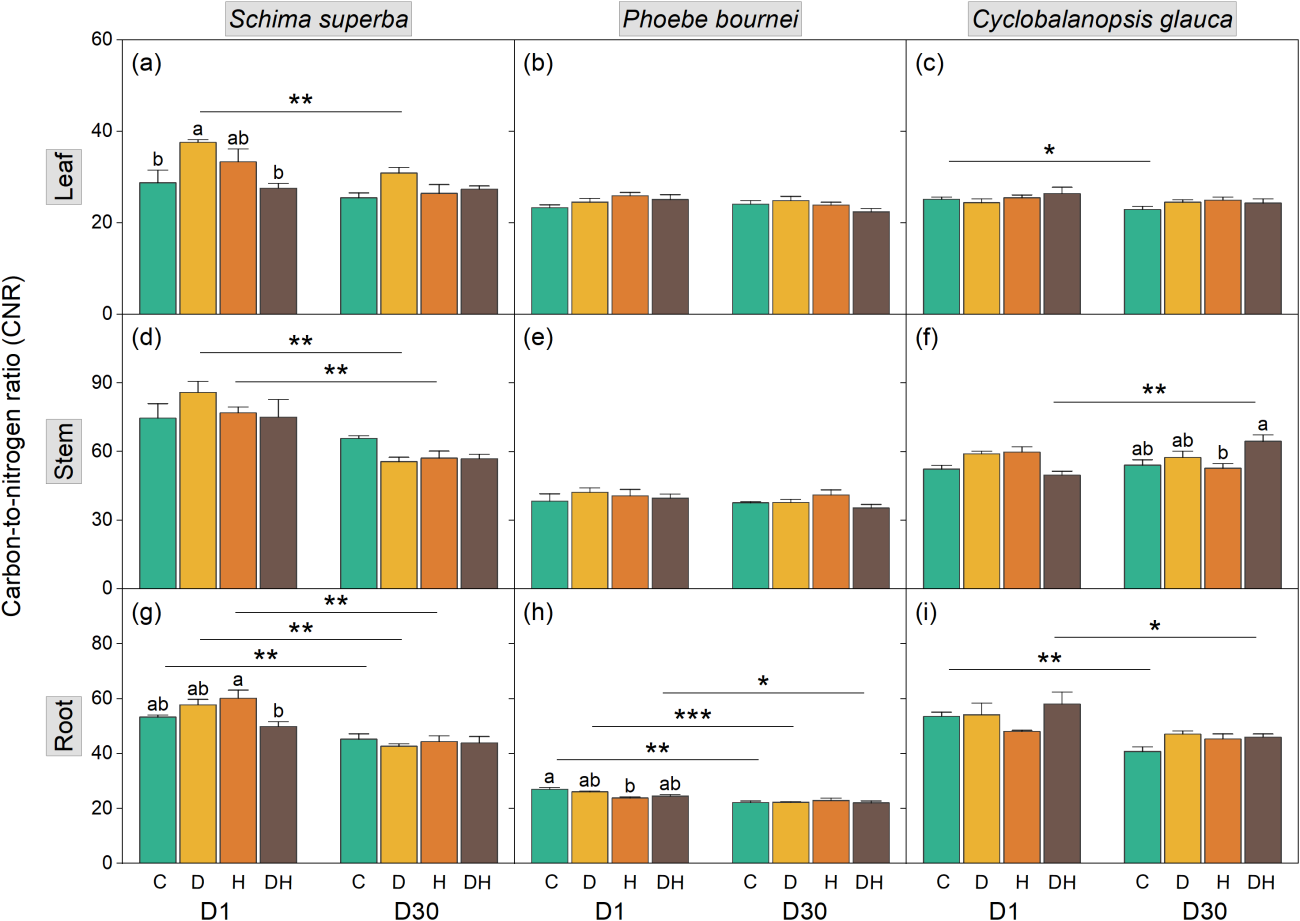
Dynamics of carbon to nitrogen ratio (CNR) in leaf **(a–c)**, stem **(d–f)**, and root tissues **(g–i)** of three subtropical tree species during rehydration. Seedlings of *Schima superba*, *Phoebe bournei*, and *Cyclobalanopsis glauca* were analyzed at 1 day (D1) and 30 days (D30) after rewatering following stress treatments. Treatments: C, control; D, water deficiency; H, heatwave; DH, combined stress. Different lowercase letters indicate significant differences between treatment groups within the same time point (*P* < 0.05). Asterisks denote significant differences for a specific treatment between D1 and D30 (*P < 0.05; **P < 0.01; ***P < 0.001).

Stress treatments (H, D, and DH) significantly influenced total carbon (TC), total nitrogen (TN), and the C:N ratio (CNR) across the three species, though the magnitude of these effects was highly dependent on the specific organ and recovery duration (**Supplementary Table 1**). A temporal shift in sensitivity was observed: significant alterations in TC were primarily delayed until the 30-day recovery period (D30) (**Figure 1**), whereas impacts on TN and CNR were most pronounced immediately after stress (D1) and generally dissipated by D30 (**Figures 2** and **3**).

Specifically, no significant short-term effects on TC were detected across any organ or species at D1. However, by D30, H and DH treatments significantly reduced stem and root TC in *S. superba* (*P* < 0.05; **Figures 1d, g**). In *P. bournei*, root TC exhibited a temporal decline from D1 to D30 across all groups, but without significant inter-treatment differences. In *C. glauca*, the D treatment resulted in the lowest root TC levels by D30 (*P* < 0.05; **Figure 1i**).

Organ TN responses were primarily concentrated at the D1 stage (**Figure 2**). In *S. superba*, water deficiency (D) significantly reduced leaf TN at D1 (*P* < 0.05), returning to control levels by D30 (**Figure 2a**). In *P. bournei*, H stress significantly increased root TN at D1 (**Figure 2h**). In *C. glauca*, stem TN decreased under D stress at D1 but recovered by D30; conversely, the DH treatment induced a significant reduction in stem TN that persisted until D30 (**Figure 2f**).

Consistent with these shifts, the CNR exhibited significant fluctuations at D1 (**Figure 3**). Drought increased leaf CNR and heatwaves increased root CNR in *S. superba* at D1 (**Figures 3a, g**). In *P. bournei*, significant CNR alterations were limited to the root under H stress at D1 (**Figure 3h**), while significant changes were observed in *C. glauca* stem CNR by D30 (**Figure 3i**).

**Figure 4:**
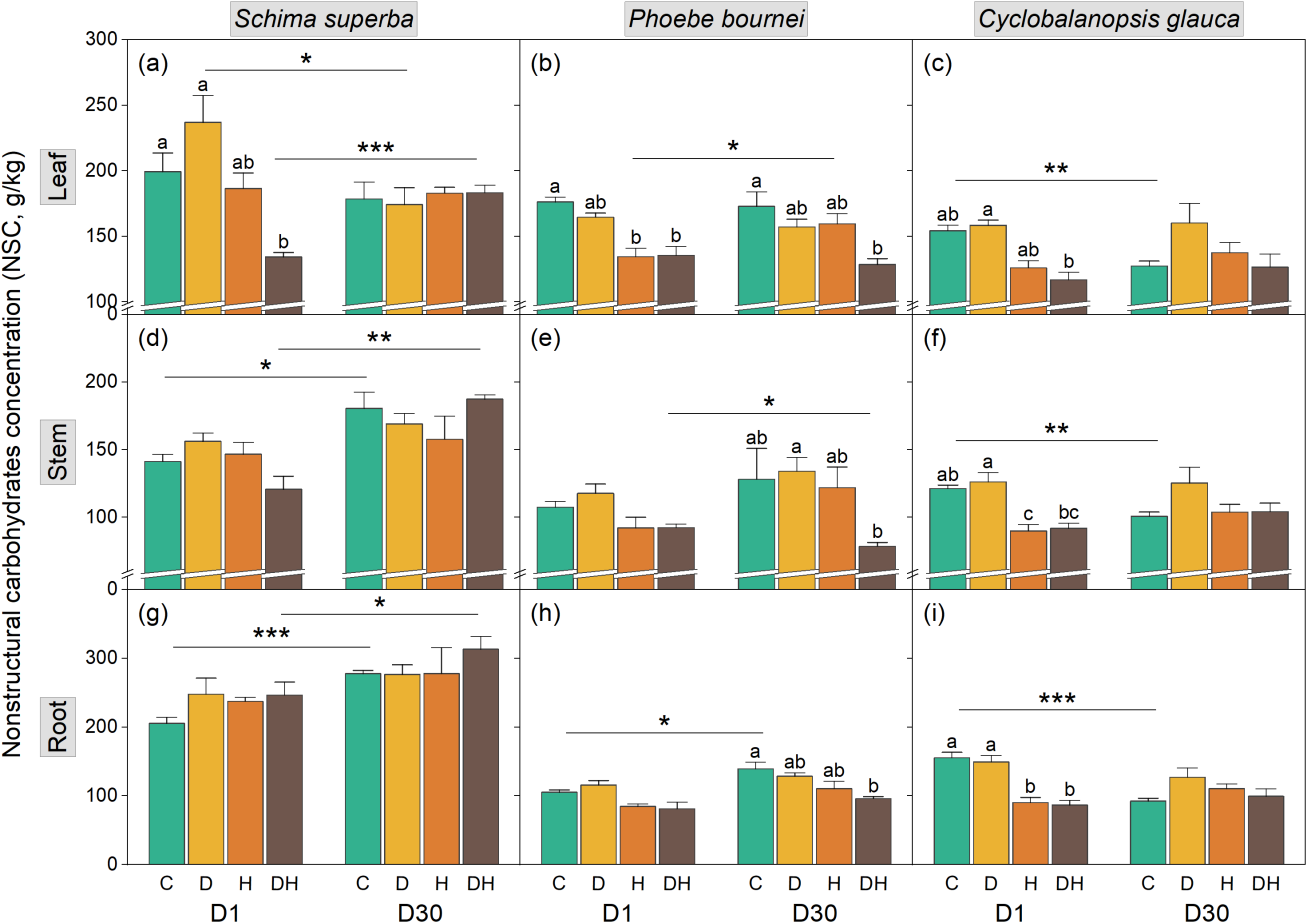
Dynamics of non-structural carbohydrate (NSC) concentration in **(a–c)**, stem **(d–f)**, and root tissues **(g–i)** of three subtropical tree species during rehydration. Seedlings of *Schima superba*, *Phoebe bournei*, and *Cyclobalanopsis glauca* were analyzed at 1 day (D1) and 30 days (D30) after rewatering following stress treatments. Treatments: C, control; D, water deficiency; H, heatwave; DH, combined stress. Different lowercase letters indicate significant differences between treatment groups within the same time point (*P* < 0.05). Asterisks denote significant differences for a specific treatment between D1 and D30 (*P < 0.05; **P < 0.01; ***P < 0.001).

Stress treatments significantly influenced the non-structural carbohydrate (NSC) concentrations of the seedlings (**Supplementary Table 1**; **Figure 4**). In *S. superba*, the impact was limited to the leaves, where DH treatment resulted in lower NSC concentrations at D1; this effect was not significant by D30 (**Figure 4a**). Similarly, *P. bournei* exhibited lower leaf NSC under H and DH treatments at D1. By D30, the DH treatment resulted in significantly lower NSC levels across all organs (leaf, stem, and root, *P* < 0.05, **Figures 4b, e, h**). In contrast, while H and DH treatments significantly reduced NSC concentrations across all organs in *C. glauca* at D1, these concentrations recovered to control levels by D30 (**Figures 4c, f, i**). Across species, leaf NSC showed the largest treatment-induced changes at D1, particularly under H and DH. The timing of whole-plant NSC responses differed: significant changes occurred at D1 in *C. glauca* but were delayed until D30 in *P. bournei*.

### 3.2 Principal components and correlations of C and N related indexes in different organs of three tree species seedlings under environmental stress

**Figure 5:**
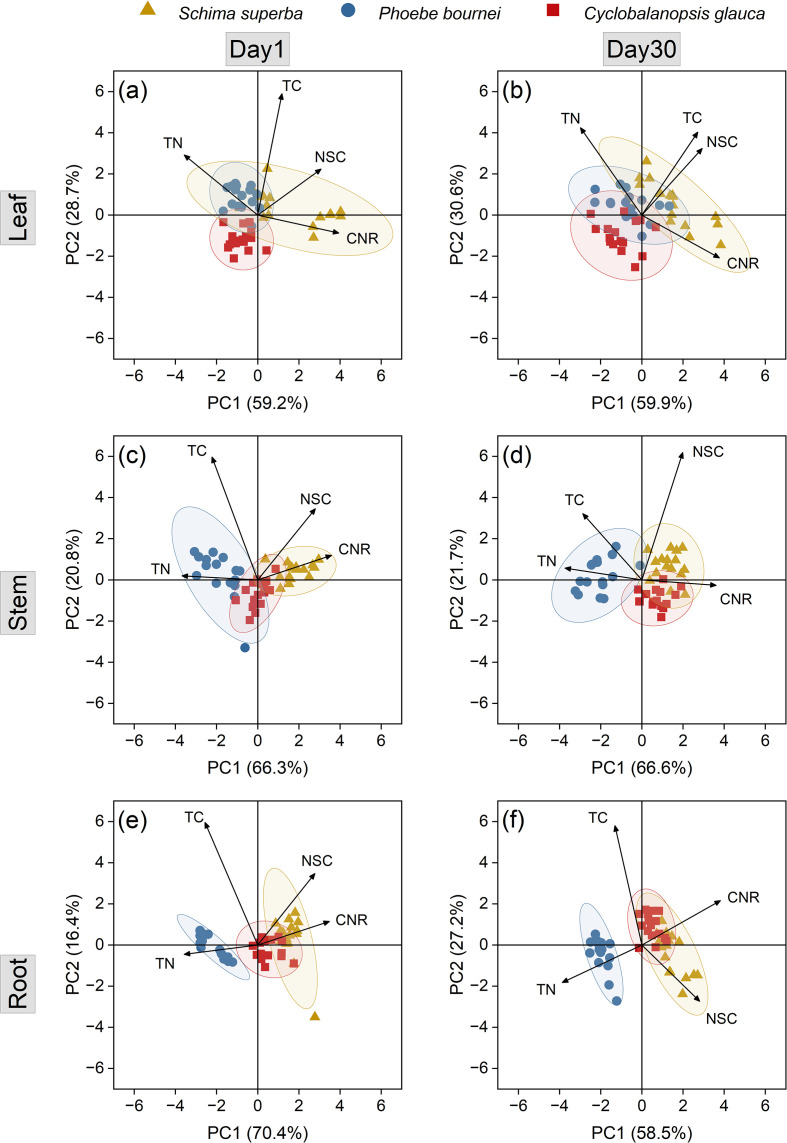
Principal component analysis (PCA) of stoichiometric and carbohydrate traits across different organs. The plots illustrate multivariate separation based on total carbon (TC), total nitrogen (TN), carbon-to-nitrogen ratio (CNR), and non-structural carbohydrate (NSC) concentrations in leaves **(a, b)**, stems **(c, d)**, and roots **(e, f)** at D1 and D30 of the rehydration phase. Ellipses represent 95% confidence intervals for each species: *Schima superba* (triangles), *Phoebe bournei* (circles), and *Cyclobalanopsis glauca* (squares).

Principal component analysis (PCA) showed separation in the multivariate space defined by stoichiometric and carbohydrate traits, which differed by organ and species (**Figure 5**). The first principal component (PC1) accounted for over 58% of the variance. From D1 to D30, the metabolic structures of leaves, stems, and roots remained stable within species, but species differences were evident. In leaves, *S. superba* clusters were separated along PC1, while those of *P. bournei* and *C. glauca* overlapped. In stems and roots, clusters for *S. superba* and *C. glauca* overlapped, while *P. bournei* was separated along PC1. The loadings of PC1 showed that this axis was closely associated with leaf CNR, and with both TN and CNR in stems and roots.

**Figure 6:**
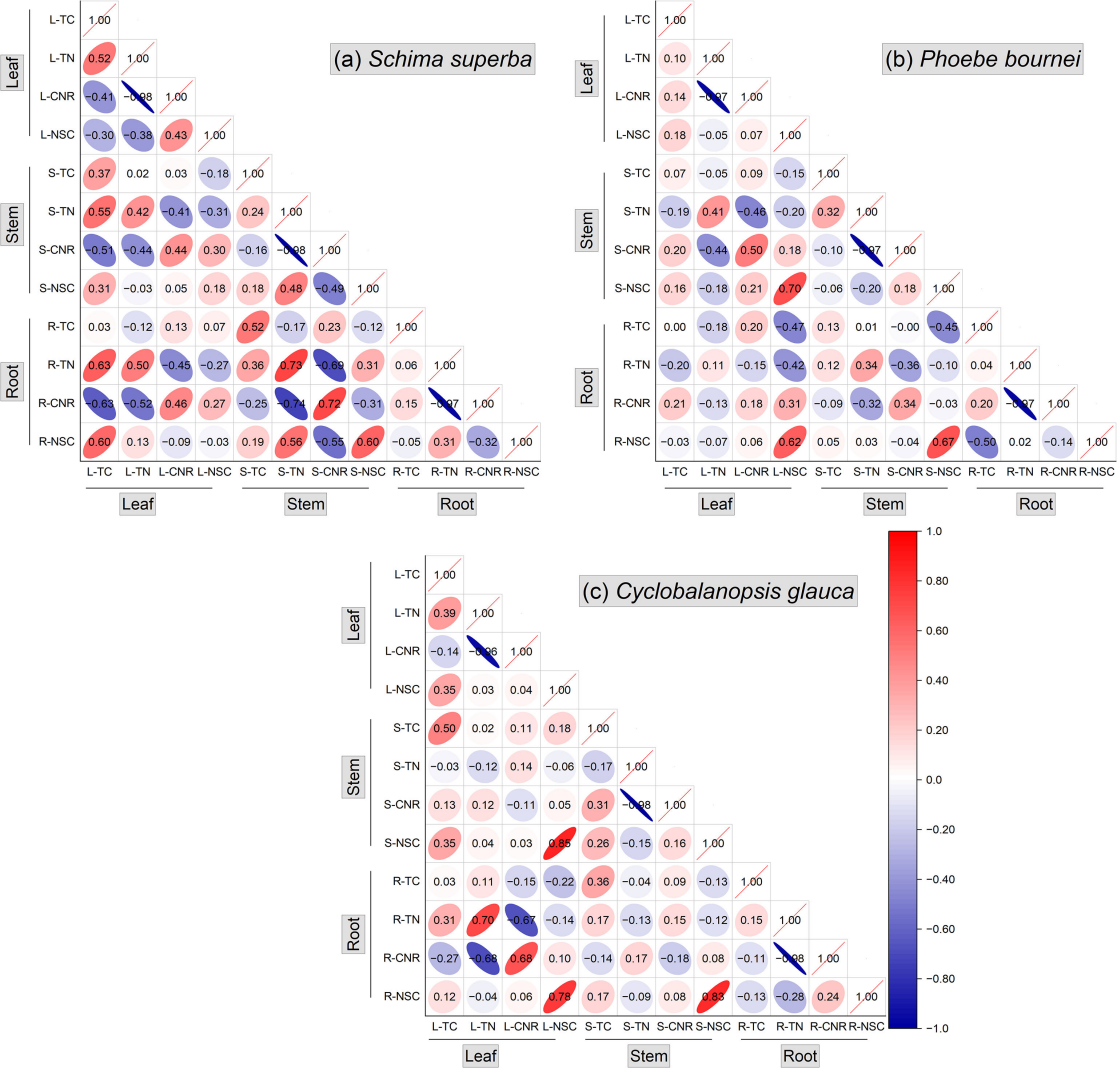
Pearson correlation matrices of carbon and nitrogen related traits across organs. Correlation coefficients are shown for leaf (L), stem (S), and root (R) tissues in *S. superba* **(a)**, *P. bournei* **(b)**, and *C. glauca* **(c)**. Traits include: TC (total carbon), TN (total nitrogen), CNR (carbon-to-nitrogen ratio), and NSC (non-structural carbohydrate content). Blue and red colors indicate negative and positive correlations, respectively.

Correlation analysis further elucidated the integrated nature of the seedlings’ stress responses, revealing significant correlations across various plant organs. Across all species, TN and CNR in all organs were significantly negatively correlated (correlation coefficient < -0.97). For *S. superba*, stem and root CNR were closely negatively related, and CNR in these organs was correlated with leaf TC and TN. TC and TN in *S. superba* were also correlated between organs (**Figure 6a**). Such inter-organ relationships were less pronounced in *P. bournei* (**Figure 6b**). In *C. glauca*, leaf and root TN and CNR were partly correlated (**Figure 6c**). Positive correlations among NSC concentrations across leaves, stems, and roots were observed in *P. bournei* and *C. glauca*, indicating a high degree of whole-plant coordination (**Figures 6b, c**) whereas only stem and root NSC were closely related in *S. superba* (**Figure 6a**).

### 3.3 Stress impacts on inter-organ distribution and temporal recovery of three tree species

**Figure 7:**
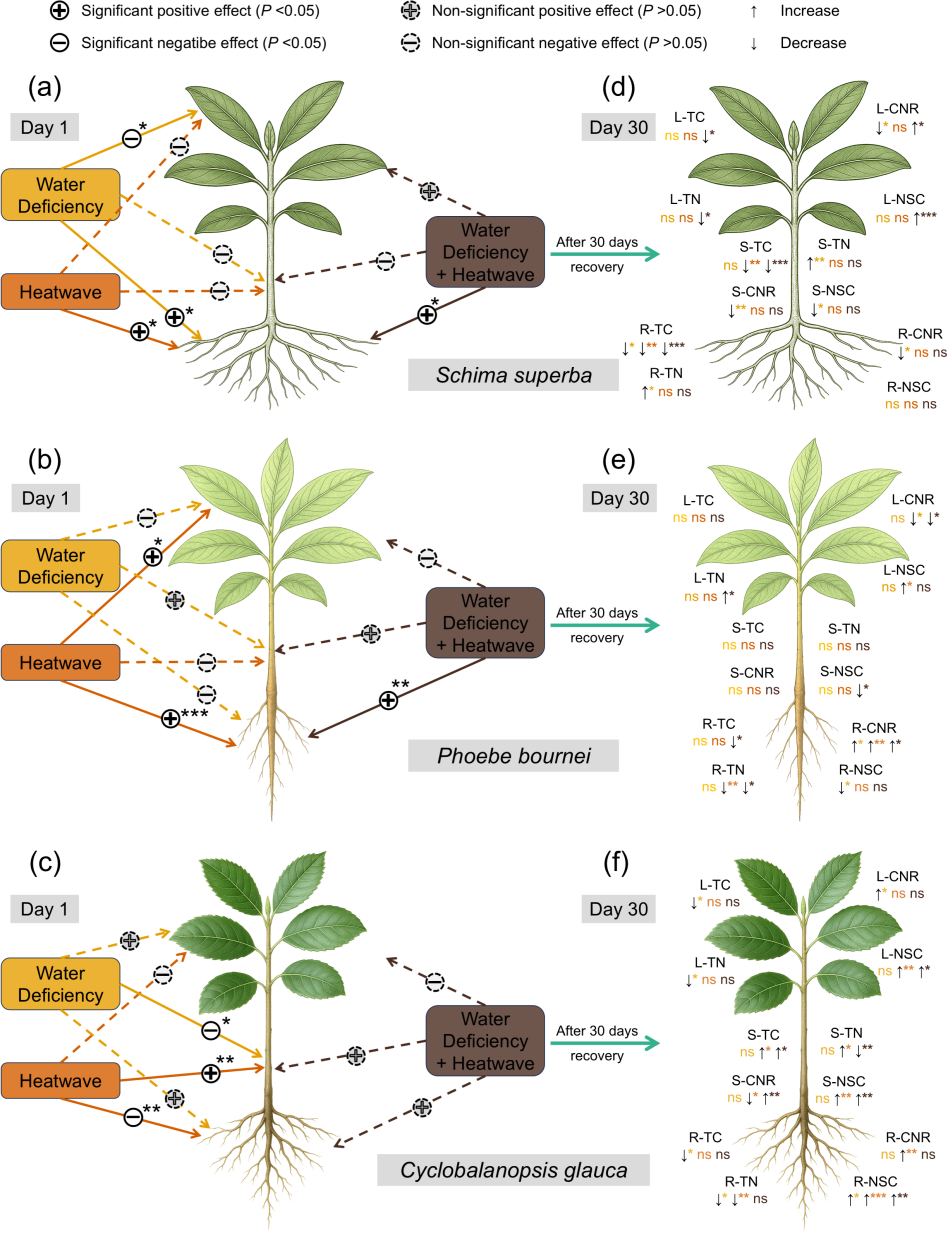
Conceptual diagram of organ-specific stress responses and recovery trajectories. **(a–c)** Direct effects of drought (D), heatwave (H), and combined stress (DH) on metabolic indices at D1, quantified via piecewise structural equation modeling (SEM). Solid and dashed lines represent significant (*P* < 0.05) and non-significant (*P* > 0.05) paths, respectively. **(d–f)** Net changes in stoichiometry and NSC after 30 days of recovery (D30) compared to D1. Up and down arrows indicate significant increases and decreases (*P* < 0.05); "ns" denotes no significant change. Significance levels: *P < 0.05; **P < 0.01; ***P < 0.001.

Structural equation modeling (SEM) quantified the direct effects of stress treatments on the metabolic status of leaves, stems, and roots at D1 (**Figure 7**). In *S. superba*, all stress treatments (D, H, and DH) exerted significant positive effects on root metabolic indices, whereas water deficiency (D) exerted a significant negative effect on leaf performance (**Figure 7a**). In *P. bournei*, heatwaves (H) showed significant positive pathways to both leaf and root metabolism, while the combined stress (DH) primarily exerted a positive impact on the roots (**Figure 7b**). For *C. glauca*, water deficiency (D) significantly negatively affected stems, while heatwaves (H) influenced stems and had a significant negative pathway to roots (**Figure 7c**).

A comparative analysis between D1 and D30 revealed treatment-specific recovery patterns and legacy effects. In *S. superba*, by D30, the significant reductions observed under H stress were largely confined to the total carbon (TC) of stems and roots compared to D1. Under combined stress (DH), persistent differences remained most evident in leaves, accompanied by alterations in stem and root TC, yet leaf NSC showed a significant recovery (**Figure 7d**). In *P. bournei*, treatment effects on recovery and nutrient distribution were primarily manifested in roots and leaves rather than stems; this was characterized by a significant elevation in root CNR contrasted with a reduction in leaf CNR (**Figure 7e**). For *C. glauca*, legacy effects on nutrient distribution were more pronounced under H and DH treatments than under D. Specifically, under H stress, notable distinctions were observed in stems and roots, driven by significantly increased CNR rather than NSC. In contrast, the combined stress (DH) exerted major legacy effects on stems—characterized by significantly elevated CNR and NSC—while concurrently increasing NSC pools in both leaves and roots (**Figure 7f**).

## 4 Discussion

### 4.1 Combined stress of water deficiency and heatwave triggers synergistic effects on C-N stoichiometry of subtropical tree seedlings

This study demonstrates that the combined stress of water deficiency and heatwaves exerts a profound synergistic impact on the C and N stoichiometry of subtropical tree seedlings, surpassing the additive effects of individual stressors. Our findings reveal that maintaining C-N homeostasis is not merely a stress response but a decisive determinant of long-term resilience. Previous research has established that the combined impact of heat and drought on plant growth and productivity is significantly more severe than that of either stressor applied individually (De Boeck et al., 2011; Gong and Hao, 2023). While high temperature primarily drives heatwave-induced stress, abrupt thermal shifts accelerate soil evaporation and plant transpiration, leading to declining soil water potential and restricted root water supply (Hussain et al., 2024; Qu et al., 2024a). Concurrently, elevated atmospheric vapor pressure deficit (VPD) triggers stomatal closure, inducing “physiological drought” (Drake et al., 2018; Wang et al., 2025a). Given that physiological responses to heat and drought involve distinct mechanisms, plant strategies under combined “dry-heat” stress cannot be predicted by simply aggregating responses to individual stressors (Zhang and Sonnewald, 2017; Zscheischler et al., 2020).

Our results underscore this complexity, with synergistic effects being more acutely reflected in NSC dynamics than in TC, TN, or CNR. Compared to individual stressors, the combined treatment (DH) induced a significantly greater reduction in organ-specific NSC pools (**Figure 4**), while TC, TN, and CNR remained relatively stable (**Figures 1**-**3**). This pronounced synergistic impact is characterized by a “dual-deficit” mechanism: the simultaneous cessation of carbon acquisition and the accelerated depletion of metabolic reserves. This pronounced synergistic impact suggests a “dual-deficit” mechanism, wherein carbon acquisition may cease while metabolic reserves are simultaneously depleted. Under combined stress, seedlings prioritize hydraulic safety by maintaining stomatal closure to prevent xylem cavitation, inevitably leading to a collapse of photosynthetic carbon assimilation (Qu et al., 2020; Guo et al., 2024). Concurrently, high temperatures exponentially increase maintenance respiration (Crous et al., 2022) and the metabolic costs associated with synthesizing heat-shock proteins and antioxidants (Yurina, 2023; Mukhopadhyay et al., 2024). This creates a severe negative carbon balance—termed a “metabolic debt”—wherein existing starch reserves in stems and roots are rapidly hydrolyzed into soluble sugars to sustain basal survival (McDowell et al., 2019; Wang and Wang, 2025). The temporal manifestation of this debt appears species-specific; notably, significant NSC depletion in *S. superba* and *C. glauca* occurred primarily at D1 rather than D30 (**Figure 4**), suggesting immediate metabolic mobilization to counter acute stress. Furthermore, drought-induced increases in phloem sap viscosity likely impair the source-to-sink transport of these dwindling carbohydrates (Hesse et al., 2018; Nawaz et al., 2025). Consequently, combined stress drives plants toward carbon starvation thresholds more rapidly than individual stressors, leaving substantial “legacy effects” that hinder physiological recovery even after stress alleviation (Robbins et al., 2024). This is particularly evident in *P. bournei*, where significant NSC variation emerged only at D30 (**Figure 4**). This delayed response indicates a protracted metabolic crisis, suggesting that different tree species employ divergent strategies in managing their “metabolic capital,” leading to varying degrees of resilience.

### 4.2 Tree species organ-specific allocation and source-sink coordination response to water deficiency and heatwaves

Our results reveal that the resistance of subtropical tree seedlings to combined dry-heat stress relies on a strict spatiotemporal division of labor among organs (**Figure 7**). The distinct response trajectories of C, N, and NSC across leaves, stems, and roots highlight a coordinated strategy that balances immediate hydraulic survival against long-term metabolic recovery.

Leaves exhibited the highest sensitivity to acute stress (D1), functioning as the primary site for metabolic modulation. As evidenced by the drastic reduction in foliar NSC under DH treatment (**Figure 4**) and the significant negative regulatory pathway in the SEM analysis for *S. superba* (**Figure 7a**), plants appear to adopt a “sacrificial” strategy during the acute phase (Qu et al., 2020). By suppressing foliar anabolic activity and rapidly mobilizing starch reserves into soluble sugars, plants maintain osmotic potential while minimizing respiratory burden (Scafaro et al., 2021; Singh et al., 2025). Crucially, the capacity for recovery defines resilience; while S. superba and C. glauca restored leaf NSC levels by D30, *P. bournei* failed to do so (**Figure 4**). This suggests that foliar metabolic plasticity—the ability to switch from downregulation back to assimilation—is a prerequisite for surviving the “legacy effects” of combined stress (Ruehr et al., 2019; Qu et al., 2024a).

In contrast to leaves, the root system functioned as a priority sink receiving resource allocation during the stress event. The SEM results consistently showed positive regulatory pathways from stress treatments to root indices at D1 (**Figures 7a, b**), reflecting an active investment to maintain hydraulic integrity and deep-water acquisition (Johnson et al., 2014; Qu et al., 2024b). However, this prioritization incurs a “delayed physiological cost.” While TN and CNR responses were immediate, significant reductions in root TC were delayed until D30 (**Figure 1**). This temporal lag indicates that the high respiratory demand of heatwaves eventually depletes structural or storage carbon pools in the roots (Rehschuh et al., 2024). The specific vulnerability of *P. bournei* is highlighted by the “root-leaf decoupling” observed at D30 (**Figure 7e**), where root CNR remained elevated while leaf CNR declined. This stoichiometric mismatch suggests a failure in source-sink transport, preventing the replenishment of belowground carbon costs incurred during the stress (Kou-Giesbrecht and Menge, 2019; Trugman et al., 2019; Gessler and Zweifel, 2024).

Stems played a critical, species-specific role as a resource buffer and transport hub (Martínez-Vilalta et al., 2016). Our legacy analysis revealed that in *C. glauca*, combined stress induced significant positive legacy effects in the stem (elevated CNR and NSC at D30; **Figure 7f**). This indicates that *C. glauca* utilizes the stem as a capacitor, sequestering resources to buffer the physiological disconnect between the dormant canopy and the stressed root system (Hesse et al., 2018). Correlation analysis further supports this, showing high whole-plant NSC coordination in *C. glauca* (**Figure 6c**). Conversely, the absence of significant stem regulation in *P. bournei* likely contributed to its systemic metabolic collapse, underscoring that a functional stem buffer is essential for resilience against the synergistic impacts of dry-heat stress (Trifilò et al., 2019; Li et al., 2022).

### 4.3 Temporal dynamics of tree resistance and recovery response to water deficiency and heatwaves: from acute resistance to metabolic legacies

The impact of environmental stress on tree seedlings is not confined to the duration of the event; it often manifests as “legacy effects”—persistent physiological deviations that continue to shape C and N dynamics long after rewatering (Ruehr et al., 2019; Qu et al., 2024a; Wang et al., 2025b). Our temporal analysis reveals that the recovery of C, N, NSC, and CNR is highly organ-specific and contingent upon the coordination of whole-plant metabolic networks. Specifically, we observed a distinct temporal decoupling in regulation, highlighting that resistance to combined dry-heat stress relies on a hierarchical resource reallocation strategy during the acute phase (D1). Immediately following stress, seedlings prioritized hydraulic safety by sacrificing foliar metabolic performance—manifested as sharp reductions in leaf NSC and significant fluctuations in N stoichiometry—while maintaining root metabolic stability, as evidenced by positive SEM pathways (**Figures 7a, b**). This defense-first allocation suggests that during acute stress, labile carbon reserves are rapidly mobilized for osmotic adjustment and maintenance respiration, while structural carbon pools (TC) remain temporarily stable (Iglesias et al., 2002; Hartmann and Trumbore, 2016). This spatiotemporal division of labor allows seedlings to mitigate immediate hydraulic failure but incurs a latent metabolic cost.

However, the true physiological toll manifests as significant “legacy effects” during the recovery phase (D30), characterized by a “metabolic debt” where structural carbon in roots is depleted to subsidize aboveground repair (Kannenberg et al., 2019; Xie et al., 2026). The capacity to resolve this debt determined species-specific resilience: *S. superba* achieved rapid recovery through foliar metabolic plasticity, whereas *P. bournei* suffered a delayed systemic collapse due to a maladaptive decoupling of root-leaf stoichiometry (**Figure 7e**), preventing effective source-sink replenishment (Ruehr et al., 2019). Conversely, *C. glauca* utilized a stem-buffering strategy—sequestering NSC and maintaining high CNR in stems (**Figure 7f**)—to bridge the gap between acute resistance and long-term hydraulic recovery (Trifilò et al., 2019; Jupa et al., 2024). Collectively, these findings underscore that post-stress resilience is contingent upon the efficient management of “metabolic capital” to buffer the temporal lag between physiological reactivation and carbon reserve replenishment.

### 4.4 Divergent interspecific response and adaptive plasticity of three subtropical to water deficiency and heatwaves

To summarize, our results indicate that the three subtropical tree species exhibited divergent evolutionary trajectories in managing their “metabolic capital” during the transition from resistance to recovery. These differences reflect distinct trade-offs between structural investment, hydraulic safety, and metabolic plasticity, which can be categorized into three adaptive models.

*S. superba* displayed the most effective resilience strategy, characterized by high metabolic plasticity. The SEM analysis (**Figures 7a, d**) revealed a dynamic shift in resource prioritization: an immediate “defense-first” allocation to roots at D1, followed by a rapid “source-restoration” allocation to leaves at D30. This species effectively utilized the recovery window to correct the foliar CNR imbalance and fully restore NSC levels (**Figure 4a**). Such physiological re-priming maintains efficient N utilization and flexible source-sink transport (Rehschuh et al., 2021), allowing *S. superba* to minimize the legacy effects of stress and ensure a swift return to carbon assimilation capabilities once environmental conditions improve (Brunn et al., 2022; Hikino et al., 2022).

In contrast to the rapid recovery of *S. superba*, *C. glauca* adopted a “conservative persistence” strategy centered on the stem. The unique accumulation of NSC and high CNR ratios in the stem at D30 (**Figure 7f**) suggests that this species utilizes the stem as a resource capacitor (Trifilò et al., 2019; Nawaz et al., 2025). Rather than investing immediately in new canopy growth, *C. glauca* sequesters metabolites in xylem parenchyma. This strategy prioritizes long-term hydraulic safety over short-term vegetative expansion, as high stem NSC concentrations are critical for maintaining osmotic pressure and facilitating the repair of stress-induced embolism (Jupa et al., 2024). This hoarding behavior acts as a buffer against environmental stochasticity, enhancing survival at the expense of rapid recovery rates.

*P. bournei* exhibited the highest vulnerability, driven by a maladaptive root-leaf decoupling. The persistence of elevated root CNR contrasted with declining leaf CNR at D30 (**Figure 7e**) indicates a preferential investment in structural carbon (e.g., cell wall lignification) in the roots, likely to prevent water efflux (Hartmann et al., 2020). However, this structural rigidity appears to come at the cost of metabolic mobility (Dominguez et al., 2022). Unlike *S. superba*, *P. bournei* failed to remobilize these root reserves to support the recovering canopy, leading to systemic carbon starvation (**Figure 4h**) and a breakdown in the source-sink continuum (McDowell et al., 2022). This suggests that *P. bournei* lacks the physiological flexibility to cope with the synergistic intensity of dry-heat stress, making it particularly susceptible to mortality under future climate scenarios.

Collectively, these findings demonstrate that resilience to combined dry-heat stress is not solely determined by the magnitude of initial damage, but by the flexibility of inter-organ coordination. The contrasting fates of *S. superba* (recovery), *C. glauca* (persistence), and *P. bournei* (decline) highlight that the ability to dynamically switch between “structural defense” and “metabolic transport” is the decisive factor in tree survival in a warming world.

### 4.5 Study limitations

Several limitations of this study should be acknowledged. First, gas exchange parameters (including photosynthetic rate, respiration rate, and stomatal conductance), hydraulic functional traits, and plant growth rates were not directly measured. As a result, our inferences regarding photosynthetic assimilation, respiratory consumption, and potential carbon starvation mechanisms are derived solely from the observed dynamics of carbon, nitrogen, C:N stoichiometry, and non-structural carbohydrate (NSC) pools across plant organs. Future studies should incorporate *in-situ* measurements of gas exchange, hydraulic traits, and growth performance to provide direct physiological validation of the mechanisms proposed here. Second, this experiment was conducted on two-year-old seedlings under controlled environmental conditions. Accordingly, the adaptive strategies identified may not fully represent the responses of mature trees in natural forest ecosystems. Further field-based investigations on adult trees are necessary to assess the generalizability of our findings across different ontogenetic stages and under more complex environmental contexts.

## 5 Conclusion

In conclusion, this study elucidates that the synergistic impact of drought and heatwaves extends beyond simple resource depletion, triggering a systemic realignment of C-N stoichiometry driven by a “dual-deficit” mechanism—involving the simultaneous cessation of photosynthetic acquisition and the exponential acceleration of respiratory consumption. We identified a critical spatiotemporal division of labor as the core resistance strategy, where seedlings prioritize acute hydraulic safety through foliar metabolic downregulation (sacrifice), while incurring a delayed “metabolic debt” in root systems that manifests as significant legacy effects during the recovery phase. Ultimately, species-specific resilience is determined by the plasticity of this inter-organ network: the adaptive “physiological re-priming” of *S. superba* and the “conservative stem buffering” of *C. glauca* proved superior to the maladaptive “structural rigidity” and stoichiometric decoupling observed in *P. bournei*. These findings underscore that under future compound climate extremes, tree survival will depend less on static resistance traits and more on the dynamic flexibility of source-sink coordination to effectively manage metabolic capital against the “invisible” costs of recovery.

## Data Availability

The datasets presented in this study can be found in online repositories. The names of the repository/repositories and accession number(s) can be found below: https://doi.org/10.6084/m9.figshare.31084042.
