# Supplementary material for: Drought and heatwave synergy alters organ-level C:N stoichiometry and carbohydrate dynamics in subtropical tree seedlings
Source: Front Plant Sci. 2026 Mar 31;17:1814780. doi: 10.3389/fpls.2026.1814780 (PMC13076567; doi:10.3389/fpls.2026.1814780)
Supplement: Supplementary file 1 [file DataSheet1.docx]

***Support Material***

**Figure. S1. Temporal dynamics of daily soil water content (SWC) and soil temperature during the experimental period (August 1 to September 30, 2023).** Treatments are defined as follows: **D** (Water Deficiency): precipitation exclusion period from August 7 to September 6 (green line); **H** (Heatwave): heat simulation period from September 2 to September 6 (red line); **DH** (Combined Stress): precipitation exclusion from August 7 followed by concurrent heat stress from September 2 to September 6. The recovery period commenced on September 7 (rewatering) and continued until the end of the experiment.


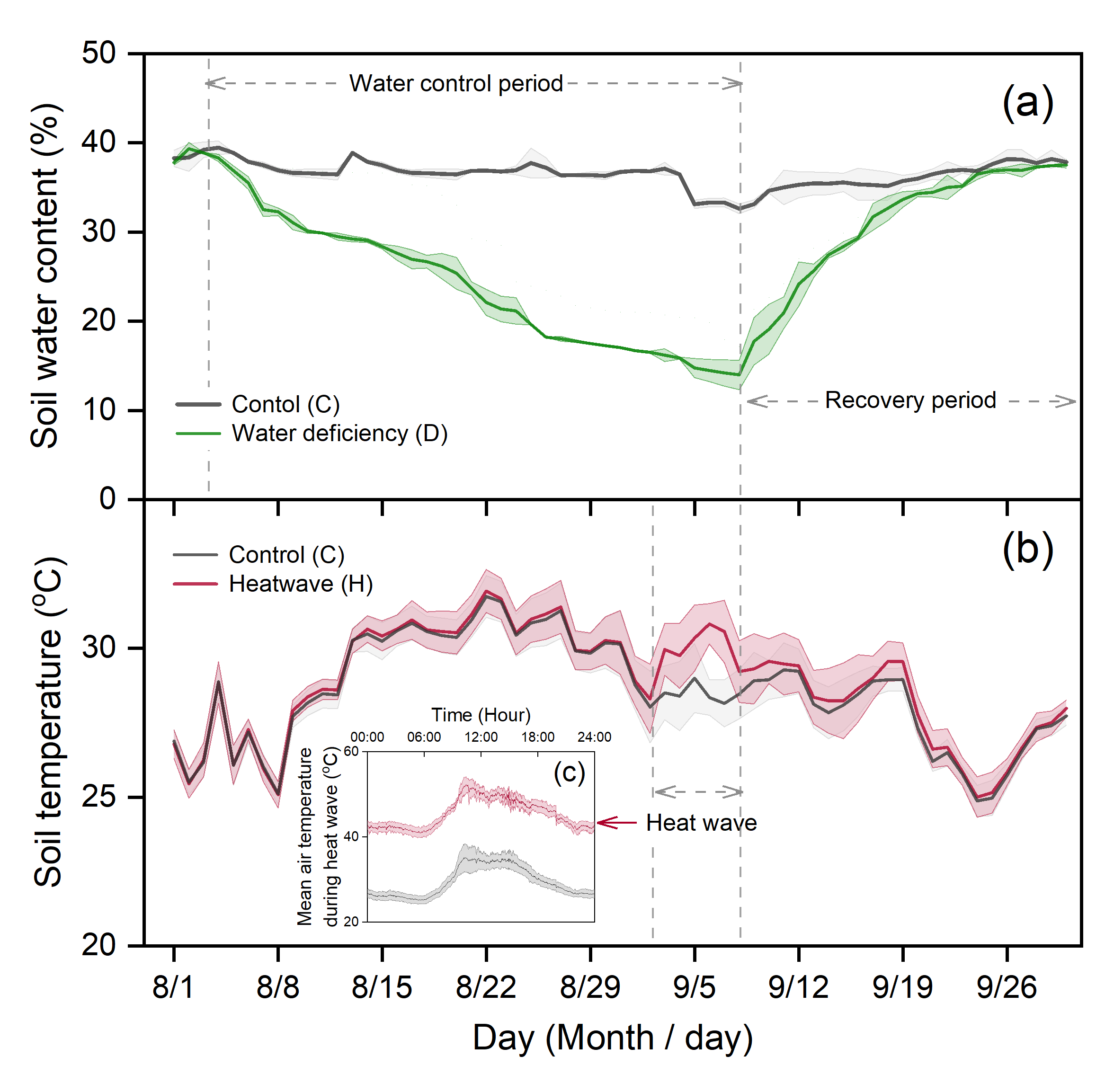


**Table S1. Summary of four-way factorial ANOVA results for total carbon (TC), total nitrogen (TN), carbon-to-nitrogen ratio (CNR), and non-structural carbohydrates (NSC).** The table presents the main and interactive effects of Species, Organ, Treatment, and Sampling Time on the physiological traits of *Phoebe bournei*, *Schima superba*, and *Cyclobalanopsis glauca* seedlings.

|  | df | C concentration (g/kg) | | N concentration (g/kg) | | C:N ratio | | NSC concentration (g/kg) | |
| --- | --- | --- | --- | --- | --- | --- | --- | --- | --- |
|  |  | F | P | F | P | F | P | F | P |
| Species (a) | 2 | 122.969 | **＜0.001** | 836.869 | **＜0.001** | 558.890 | **＜0.001** | 446.639 | **＜0.001** |
| Organs (b) | 2 | 95.342 | **＜0.001** | 1676.029 | **＜0.001** | 1042.567 | **＜0.001** | 87.540 | **＜0.001** |
| Treatments (c) | 3 | 0.809 | 0.490 | 6.996 | **＜0.001** | 3.968 | **0.009** | 24.680 | **＜0.001** |
| Time (d) | 1 | 4.847 | **0.029** | 111.137 | **＜0.001** | 114.141 | **＜0.001** | 19.515 | **＜0.001** |
| a×b | 4 | 88.068 | **＜0.001** | 139.517 | **＜0.001** | 103.975 | **＜0.001** | 91.714 | **＜0.001** |
| a×c | 6 | 0.886 | 0.506 | 2.469 | **0.025** | 2.652 | **0.017** | 3.965 | **＜0.001** |
| a×d | 2 | 9.286 | **＜0.001** | 10.017 | **＜0.001** | 37.091 | **＜0.001** | 11.564 | **＜0.001** |
| b×c | 6 | 0.729 | 0.627 | 2.501 | **0.023** | 0.289 | 0.942 | 1.332 | 0.244 |
| b×d | 2 | 11.130 | **＜0.001** | 5.520 | **0.005** | 10.987 | **＜0.001** | 8.062 | **＜0.001** |
| c×d | 3 | 2.874 | **0.037** | 0.445 | 0.721 | 3.148 | **0.026** | 6.139 | **＜0.001** |
| a×b×c | 12 | 3.373 | **＜0.001** | 3.671 | **＜0.001** | 1.446 | 0.147 | 2.152 | **0.015** |
| a×b×d | 4 | 4.897 | **＜0.001** | 2.685 | **0.032** | 13.286 | **＜0.001** | 6.266 | **＜0.001** |
| a×c×d | 6 | 8.311 | **＜0.001** | 3.840 | **0.001** | 3.279 | **0.004** | 7.174 | **＜0.001** |
| b×c×d | 6 | 1.165 | 0.326 | 2.468 | **0.025** | 2.351 | **0.032** | 0.745 | 0.614 |
| a×b×c×d | 12 | 0.371 | 0.973 | 1.965 | **0.029** | 2.661 | **0.002** | 1.190 | 0.292 |

Bold numbers indicate significant differences (*P* < 0.05)
